# The Origin and Advancement of Cardiovascular Physiology in Brazil: The Contribution of Eduardo Krieger to Research Groups

**DOI:** 10.3389/fphys.2016.00135

**Published:** 2016-04-19

**Authors:** Elisardo C. Vasquez

**Affiliations:** ^1^Laboratory of Translational Physiology, Federal University of Espírito SantoVitória, Brazil; ^2^Pharmaceutical Sciences Graduate Program, Vila Velha UniversityVila Velha, Brazil

**Keywords:** Eduardo Krieger, Braun Menéndez, Bernardo Houssay, cardiovascular physiology, angiotensin

## Abstract

Since 1996, symposia devoted to the discussion of advances in cardiovascular physiology have been alternately organized by Brazilian research groups, most of which were created or joined by Ph.D. trainees of Eduardo M Krieger. Therefore, as *Frontiers in Physiology* is publishing a topic devoted to the celebration of the 20th edition of the Brazilian Symposium of Cardiovascular Physiology, it is a great opportunity to talk about the contributions of Eduardo Krieger to the development of cardiovascular physiology. In this historical mini-review, first, the influence of the Argentinian group of Bernardo Houssay and Braun Menéndez on cardiovascular physiology in Brazil is discussed. Second, the contribution of Eduardo Krieger to the creation of several of those groups and to the development of science and technology is reviewed. Finally, the origin and consolidation of the group of Vitoria is highlighted as an example of a research group that was influenced by the University of Sao Paulo-Faculty of Medicine of Ribeirao Preto and has trained hundreds of Master and Ph.D. students in the area of cardiovascular research.

## Introduction

The scientific career of Eduardo Krieger has been described through interviews that have been conducted in Portuguese and translated to English (Moura and Zorzetto, 2012) and was recently commented on in a book about the career of his master Braun Menéndez (Krieger, 2015). Now, thanks to the editors of this special topic devoted to celebrate the 20th Brazilian Symposium of Cardiovascular Physiology, there is an opportunity to demonstrate in *Frontiers in Physiology* our gratitude to those who have immensely contributed to the creation and consolidation of research groups in cardiovascular physiology in Brazil.

**Figure 1:**
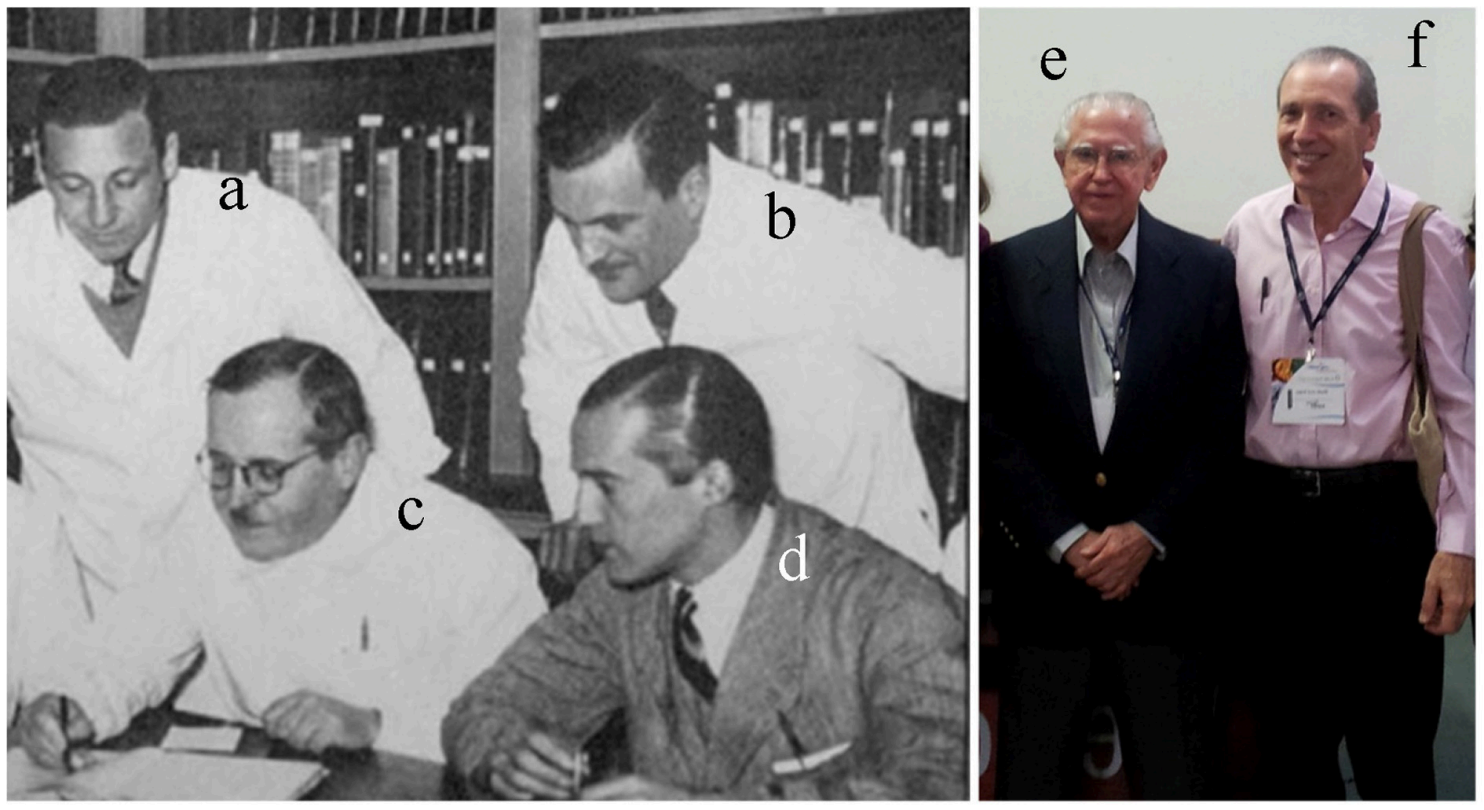
**The Argentinian group of cardiovascular physiology (1940)**. Taquini **(a)**, Braun Menéndez, co-discoverer of angiotensin **(b)**, Houssay **(c)**, and Leloir **(d)**, the two Nobel Prize laureates. The master of Brazilian cardiovascular physiology and hypertension, Eduardo Krieger **(e)**, and his disciple Elisardo Vasquez **(f)** (2014).

This mini-review encompasses the point of view of a senior investigator in cardiovascular physiology who received his Ph.D. research training from Eduardo Krieger (Figures 1e,f) at the University of Sao Paulo-Faculty of Medicine of Ribeirao Preto (FMRP-USP), focused on the contribution of his master and the FMRP-USP to the creation of various research groups in cardiovascular physiology. Moreover, our knowledge of the beginning of cardiovascular physiology research in Brazil comes from the chair of the Department of Physiology of the FMRP-USP, Miguel R. Covian, who created a group of eight Ph.D. students interested in philosophy and to talk about science and scientists, including the fascinating history of Bernardo Houssay's leadership. This mini-review is consistent with a philosophical phrase I use, “in our life and science, as much as we know where we came from, as much as we will be able to decide where to go.”

## Argentinian masters who contributed to the development of cardiovascular physiology in brazil

The beginning of cardiovascular physiology research in Brazil occurred in the middle of the past century, with the convergence of several factors. In 1947, Bernardo Houssay, the leader of a group of physiologists at the Faculty of Medicine of the University of Buenos Aires, was awarded the Nobel Prize in Physiology or Medicine. His disciples included important investigators such as the physiologist Braun Menéndez, who was one of the discoverers of angiotensin in 1940 and the cardiologist Alberto Taquini (Figures 1a–d). In 1951, two important public agencies in Brazil were created: The National Council for the Development of Science and Technology (CNPq), with the mission of supporting science and technology, and the Coordination for the Improvement of Higher Education (CAPES), with the mission of supporting science at universities.

In 1953, the physician Rubens Maciel, working at the School of Medicine of the Federal University of Rio Grande do Sul (UFRGS) in the city of Porto Alegre, acquired financial support from CAPES to establish an agreement between the School of Medicine and the Institute of Biology and Medicine Research recently created by Bernardo Houssay's group, with the goal of collaboratively training new Brazilian physiologists. Additionally, in 1953, the FMRP-USP was created and 2 years later Miguel R. Covian, from Bernardo Houssay's group, was hired to chair the Department of Physiology at that institution.

## The masters of Eduardo Krieger

In 1953, Eduardo M. Krieger, native of the State of Rio Grande do Sul, who during the last term of his medical course in Porto Alegre decided to become a cardiologist and was working at the School of Medicine with the cardiologist Rubens Maciel, met the famous Braun Menéndez and became fascinated by his enthusiasm for science and his facility for problem solving. Reciprocally, Rubens Maciel and Braun Menéndez noted the potential and scientific ability of the young Eduardo Krieger and proposed him to work on the renin-angiotensin system at the Braun Menéndez and Bernardo Houssay laboratory (1955), which was located in a large house belonging to the Braun Menéndez family. The group had left the university in protest against the military dictatorship. Eduardo Krieger described that period of his work in the laboratory of Braun Menéndez as a fascinating opportunity to interact with other famous scientists, such as Luis Leloir, who had a lab in a smaller house close to the Menéndez house and who was awarded the Nobel Prize in 1970 for his discoveries in chemistry (Figures 1a–d). In Porto Alegre, Eduardo Krieger was dedicated to work in cardiovascular physiology and experimental hypertension in collaboration with Rubens Maciel and Braun Menéndez. Then, having been recommended by Bernardo Houssay and with a grant from the Rockefeller Foundation, in 1956, he decided to pursue scientific training in the USA. There, he worked with William Hamilton at the University of Georgia, where he also had the opportunity to meet Raymond Ahlquist, who discovered the α- and β-adrenoceptors. While there and with plans to return to Porto Alegre to work in cardiology, Krieger was invited by the head of the Dept. Physiology of the FMRP-USP, Miguel Covian, to assume the position of professor and to create a cardiovascular physiology division.

## Eduardo Krieger's career at the FMRP-USP

Eduardo Krieger, now 88 years old, initiated his career as professor of cardiovascular physiology in 1957 at the FMRP-USP upon graduating from the first medical class at the institution. He has noted that he found a scientific atmosphere at the FMRP-USP that resembled what he had observed at Bernardo Houssay's and Braun Menéndez's labs. The FMRP-USP had excellent research laboratories, substantial financial support from the Rockefeller Foundation for new investigators and houses on-campus for the residence of full-time investigators. All of these favorable conditions were designed to develop research and were attractive not only to Eduardo Krieger but also to other investigators who had been invited to fill open positions in the departments of biochemistry, physiology, and pharmacology at FMRP-USP. Two years later, Krieger planned a visit by his mentor, Braun Menéndez, to develop collaborative research projects, but it was canceled due to the premature and unexpected death of the still young Braun Menéndez.

## The cardiovascular discoveries of Eduardo Krieger

A couple of years after arriving at the FMRP-USP, while waiting for the equipment he had applied for, Krieger decided to study the mechanisms by which the peripheral and central nervous systems regulate blood pressure (BP) in conditions of hypothermia. In that study, he stimulated the nerves of the cervical region in rats and casually observed that the stimulation of the central part of the vagus nerve caused both increases and decreases in BP. Then, under a higher magnification microscope, he noted that the vagus nerve was not a single nerve but included a second nerve. He observed that the stimulation of one nerve caused an increase in BP and that the stimulation of the other nerve caused a decrease in BP. After that observation, Krieger conducted systematic experiments to determine how those nerves controlled BP and concluded that it was possible to isolate the sympathetic nerve from the vagus nerve in the rat species and that the sympathetic nerve contained aortic baroreceptor fibers. Those studies led him to work on another project, which resulted in the creation of the sino-aortic denervation (SAD) model, which became known and used worldwide after its publication in *Circulation Research* in 1964 (Krieger, 1964); the article has accumulated more than 600 citations. Already training post-graduate students, Eduardo Krieger subsequently focused his studies on the baroreflex control of BP and the conditions of sustained hypertension. He and his Ph.D. students demonstrated that the baroreceptors are reset to operate at higher BP levels in hypertension and that a complete resetting occurs when the increase in the pressure threshold equals the increase in BP, which is observed after 48 h of sustained hypertension in rats (Krieger, 1989).

In 1985, after he had retired from the FMRP-USP, Krieger accepted an invitation to be a full professor and investigator at the Heart Institute (InCor) of the USP Hospital with the mission of developing integrative investigations in cardiovascular physiology and in experimental and clinical hypertension, transferring the knowledge from the bench to the bedside.

## The contribution of Eduardo Krieger to scientific organizations

The career of the tireless Eduardo Krieger was not restricted to publishing papers and supervising new investigators. He was one of three editors who, in 1981, created the important internationalized *Brazilian Journal of Medical and Biological Research*. After being president of the Brazilian Society of Physiology (1979–1985), he was the primary contributor to the creation of the Federation of Experimental Societies of Biology (FeSBE) and its first president (1985–1991). Many famous investigators working in cardiovascular physiology attended FeSBE meetings during Professor Krieger's tenure as President. Furthermore, the organization provided hundreds of opportunities for young physiologists to train at prominent universities around the world. Krieger was one of the creators and presidents of the Brazilian Society of Hypertension (1992–1994) and the Inter-American Society of Hypertension. He served as chairman of the Brazilian Academy of Sciences (1992–2007), an organization linking science to society and government; and has served as vice-chairman of the Foundation for the Development of Science of Sao Paulo (Fapesp) since 2010. Thus, he continues to play a major role in the development of science and technology throughout Brazil.

## PH.D. students trained in cardiovascular physiology by Eduardo Krieger

By the 1970s, when CAPES created the national post-graduation system, the universities were undergoing an expansion and many recently graduated individuals and hired professors were waiting for an opportunity to begin a Ph.D. degree. Unfortunately, at that time few labs were able to offer Ph.D. training in cardiovascular research. The laboratory of Eduardo Krieger was one of the most sought after and active laboratories and within a few years he had trained several Ph.D. students. Some of the graduates were hired by the Departments of Physiology and Pharmacology of the FMRP-USP and created new labs and new possibilities for training Ph.D. students in cardiovascular physiology, in collaboration with Krieger's lab. Many of the Ph.D. graduates returned to their previous positions in other universities or were rapidly hired and joined or created new cardiovascular research groups at various places. Overall, Eduardo Krieger trained more than 30 Ph.D. students and one-third of them became full professors who have been internationally recognized as important investigators and who have created or joined other groups of cardiovascular physiology throughout Brazil.

Considering the students who were supervised by Krieger at the FMRP-USP and those supervised by his disciples, more than 200 Ph.D.'s were trained by the group of Eduardo Krieger in the investigation of cardiovascular physiology. This number is markedly greater if one considers the period after his retirement, when he worked as the head of the Hypertension Unit at InCor-USP, where he trained Ph.D. students in basic science areas and clinicians to work in new fields of research, such as BP regulation during sleep and exercise and the recording of sympathetic nerve activity in physiological and clinical conditions.

## The contribution of the FMRP-USP to the creation of the vitoria group: The origin of an important research group in cardiovascular physiology

The success of the cardiovascular physiology group at the Federal University of Espirito Santo (Ufes) in Vitoria could be considered unexpected. In 1981, Ufes had only one post-graduation program and did not have a department of physiology or physiological sciences; I brought the first Wistar rats to the department for research from the FMRP-USP. On the other hand, the group became feasible because there was a resident and very active physiologist, Dalton Vassallo, who welcomed Ph.D. trainees in biochemistry (one), physiology (myself), and pharmacology (four) from the FMRP-USP and one who had a Ph.D. degree from the University of Birmingham in the UK. Additionally, the new members of the group started sharing space, material, equipment, and technicians and working together to understand the control of cardiovascular function using the rat model of SAD and rat models of hypertension. In 1989, the group of eight researchers was consolidated and was authorized by CAPES to create a Master's degree course, which was inaugurated with a Lecture from Eduardo Krieger.

At that time, my master suggested that I should go to the USA to work with Michael J. Brody at the University of Iowa, who gave me the position of visiting associate professor (1989–1991). I opted to work on the central control of BP and the rostral ventrolateral medulla (Vasquez et al., 1992). However, similar to Braun Menéndez, Brody had an abrupt death and we did not have the opportunity to plan a collaborative work with him at Ufes.

In 1991, when attending The Council for High Blood Pressure Research of the American Heart Association, in Baltimore, Eduardo Krieger told me that the Vitoria group had reached maturity and that we should attempt to create the first post-graduate program for Ph.D.'s at Ufes. We did so in 1993, and in a couple of years, that program achieved the maximum score (A) from CAPES.

In addition to his direct contribution to the creation of the Vitoria group, Krieger also had important influences on the training of members of this group by many famous cardiovascular physiologists. We highlight the collaboration between the Vitoria group and the group of Michael Brody, François Abboud, Mark Chapleau, and Alan Kim Johnson in Iowa City on two occasions; the group of Kurt Varner, Daniel Kapusta, and Louis Barker at Louisiana State University; the group of Michel Safar at Paris Descartes University; the group of Bernard Fleury at Hôpital Saint-Antoine in Paris; and the group of Virend Somers at the Mayo Clinic College of Medicine, all of which had been planned when I was working in Iowa City in two occasions (1989–1991 and 1998–2000). Other important and long-lasting scientific influences came from Michael Spyer at The University College of London Medical School in collaboration with Henrique Futuro-Neto, from Mercedes Salaices at the Universidad Autonoma de Madrid in collaboration with Dalton Vassallo and from José Krieger at InCor-USP in collaboration with José Geraldo Mill.

Interestingly, the collaborative work of Silvana Meyrelles in Chapleau's lab and of myself in Kim Johnson's lab (1998–2000) enabled us to improve our studies with addition of molecular biology techniques, gene therapy, and knockout mouse models at Ufes. Thus, we began translational research using a combination of *in vivo* and *in vitro* approaches, which culminated with the creation of the Lab of Translational Physiology, which has substantially contributed to the progress of our group. In the past 20 years, this laboratory has focused on the characterization of cardiovascular dysfunction in mouse models of atherosclerosis and hypertension, the identification of new therapies, and decreasing the time that a scientific discovery takes to reach clinical practice (Vasquez et al., 2012), as has been proposed by the societies of physiology (Seals, 2013). To date, the Vitoria group has provided outstanding training to 275 Masters and 130 Ph.D. students in cardiovascular physiology. Those Ph.D.'s have successfully filled positions in academia, either joining established groups or creating new groups.

## Conclusion

Eduardo Krieger, who was trained by Braun Menéndez, Houssay, and Hamilton, has contributed enormously to the advancement of science through the creation and leadership of scientific organizations and molding of young investigators to work in cardiovascular physiology. He was an exemplary master and taught us that as investigators, we should maximize our research efforts to contribute to better life conditions for the population.

## Author contributions

EV contributed with the conception, review design, and contents of the manuscript.

### Conflict of interest statement

The author declares that the research was conducted in the absence of any commercial or financial relationships that could be construed as a potential conflict of interest.
